# 427. Healthcare Personnel Perceived Benefit of Infection Prevention Strategies during COVID

**DOI:** 10.1093/ofid/ofab466.627

**Published:** 2021-12-04

**Authors:** Emily Sickbert-Bennett, Natalie Schnell, Shelley Summerlin-Long, Brooke Brewer, Lauren DiBiase, Lisa Stancill, Lisa Teal, David J Weber, David J Weber

**Affiliations:** 1 UNC Health Care, Chapel Hill, NC; 2 UNC Medical Center, Chapel Hill, North Carolina; 3 University of North Carolina, Chapel Hill, NC

## Abstract

**Background:**

During the COVID-19 pandemic, many infection prevention policy and practice changes were introduced to mitigate hospital transmission. Although each change had evidence-based infection prevention rationale, healthcare personnel (HCP) may have variable perceptions of their relative values.

**Methods:**

Between October-December 2020, we conducted a voluntary, anonymous, IRB-approved survey of UNC Medical Center HCP regarding their views on personal protective equipment (PPE) and hospital policies designed to prevent COVID acquisition. The survey collected occupational and primary work location data (COVID unit or not) as well as their views on specific infection prevention practices during COVID. Chi squared tests (two tailed) were used to compare differences in the proportions.

**Results:**

The overall results are displayed (Figure). Among the 694 HCP who responded to the survey, we found HCP were largely (68%) satisfied that the organization was taking all the necessary measures to protect them from COVID-19. A significantly greater proportion (14% more) of HCP (81.7% compared to 67.6%; 95% CI of difference 9.4-18.5%, P< 0.0001) agreed that all PPE was available to them compared to those who were confident that the organization was taking necessary steps for protection, highlighting that safety is more than simply availability of supplies. More than 90% felt that daily screening of patients/visitors and patient/visitor mask requirements were important for protecting them from acquiring COVID in the workplace and that wearing a mask themselves was a key intervention for protecting others. Fewer HCP (72-80%), although still a majority, perceived that eye protection and daily symptom screening for HCP were beneficial. Symptom screening for patients/visitors was perceived by 19% more HCP (90.9% compared to 72.2%; 95% CI of difference 15-23%) to be beneficial than symptom screening of HCP (P< 0.0001).

Figure. HCP Perceived Benefit of Infection Prevention Strategies during COVID

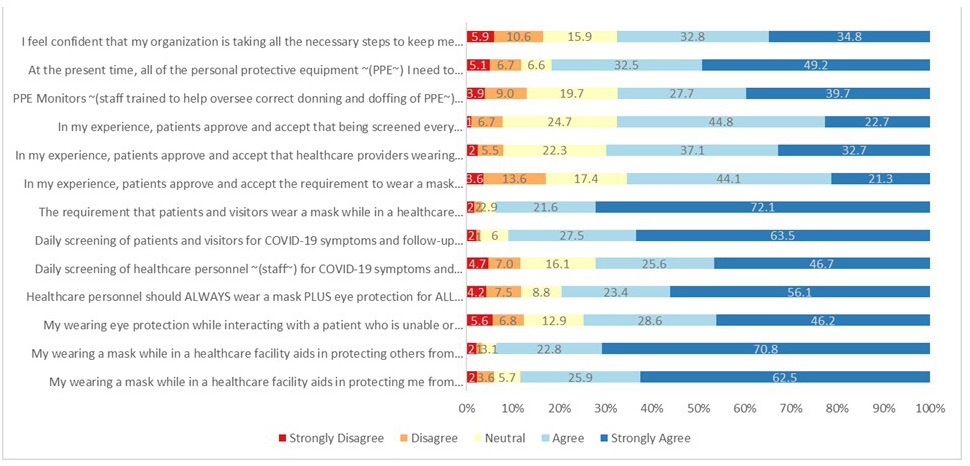

**Conclusion:**

Although infection prevention strategies were implemented based on evidence and in alignment with CDC recommendations, it is important to acknowledge that the perception and acceptance of these recommendations varied among our HCP. Compliance can only be optimized with key interventions when we seek to understand the perceptions of our staff.

**Disclosures:**

**David J. Weber, MD, MPH**, **PDI** (Consultant)

